# Changes in salt-marsh carabid assemblages after an invasion by the native grass *Elymus athericus* (Link) Kerguélen

**DOI:** 10.3897/zookeys.100.1537

**Published:** 2011-05-20

**Authors:** Anita Georges, Philippe Fouillet, Julien Pétillon

**Affiliations:** 1 U.M.R. 6553 – ‘‘Ecosystèmes, Biodiversité, Evolution’’, Université de Rennes 1, 263 Avenue du Gal Leclerc, CS74205, 35042 Rennes Cedex, France; 2 U.R.U. 420 – ‘‘Biodiversité fonctionnelle et Gestion des territoires’’, Université de Rennes 1, 263 Avenue du Général Leclerc, CS 74205, 35042 Rennes Cedex, France; 3 Evolutionary Ecology Group, University of Antwerp, Groenenborgerlaan 171, 2020 Antwerpen, Belgium

**Keywords:** Coleoptera, Carabidae, native invasive species, salt marsh, ecological indicators

## Abstract

As a result of an invasion by the native grass *Elymus athericus* (Link) Kerguélen (Poaceae) in the last 10 years, a major change in vegetation cover has occurred in salt marshes of the Mont Saint-Michel bay, Western France. The impact of such an invasion on carabid assemblages, a dominant group of terrestrial arthropods in these habitats and containing several stenotopic species, is investigated here. In our study site, carabid data are available from 1983 and 1984, allowing a comparison of species distribution ranges in salt marshes before (1983–1984) and after (2002) the *Elymus athericus* invasion. A total of 16,867 adults belonging to 40 species were caught. By considering the presence-absence of species shared between studies, we show that the invasion by *Elymus athericus* promoted the progression of non-coastal species (mainly *Pterostichus* s.l. spp.). This did however not interfere with resident species distributions, finally resulting in higher carabid species richness in the entire area. The species composition and abundances of carabid assemblages were also compared between natural and invaded stations in 2002. The main result is that abundances of some halophilic species decreased in one invaded plot (in case of *Pogonus chalceus* (Marsham 1802)) whereas the opposite pattern was observed for other species (e.g., *Bembidion minimum* (Fabricius 1792)). Invaded habitats were characterized by lower percentages of halophilic species and higher total species richness.

## Introduction

Intertidal salt marshes are ecosystems located between land and sea, undergoing periodical flooding during tides, occurring around twice a month in West-Europe. This creates some special habitat conditions, and marsh plants and animals often have special adaptations to cope with these. Salt-marsh arthropods are able to withstand floods and salinity by physiological, behavioural or morphological adaptations (e.g., Foster and Treherne 1976, Irmler et al. 2002, Pétillon et al. 2009). Salt marshes are among the rarest habitats in the world, covering less than 0.01% of the Earth’s surface (Desender and Maelfait 1999, Lefeuvre et al. 2003). In Europe, their surface strongly declined during the last decades, reinforcing the conservation interest in their original flora and fauna (Bakker et al. 2002). There is thus an urgent need to study human impacts that can either threaten (by e.g., over-grazing or habitat destruction), or enhance (by appropriate management) halophilic species in salt marshes (Goeldner-Gianella 1999, Adam 2002).

More recently, salt marshes have been invaded in many West-European sites by the nitrophilous grass *Elymus athericus* (Poaceae) (Valéry et al. 2004), probably due to increases in soil nitrogen (via the accumulation of nitrogenous compounds in the plant: Leport et al. 2006) and/or to the abandonment of agricultural practises (e.g., Esselink et al. 2000). Although *Elymus athericus* is a native species in Europe (Bockelmann and Neuhaus 1999) – usually growing in the upper parts of salt marshes – it can form dense, mono-specific stands, which corresponds to an invasion. This is likely to modify biodiversity and consequently ecosystem proprieties and functions as well as the conservation value of invaded areas (Valéry et al. 2009). Invaded areas mainly differ from natural habitats (usually dominated by *Atriplex portulacoides*, Chenopodiaceae, in ungrazed middle marshes) by their enhanced litter layer and by their higher plant cover.

According to McGoech (1998), a taxonomic group is an ecological indicator if it responds to environmental changes, stressful or not. In this study, we focussed on ground beetles (Coleoptera, Carabidae) as they are known to react quickly and strongly to changes in micro-habitat conditions. This group is thus frequently used as an indicator of human disturbances or management practices (e.g., Luff et al. 1992, Georges 1994, Sunderland and Lövei 1996, Rainio and Niemelä 2003). The assessment of human impact was conducted by comparing two conservation criteria, i.e., abundance of halophilic species and species richness, between natural and invaded stations. Species richness is widely used as a conservation target (e.g., Noss 1990, Bonn and Gaston 2005). The use of stenotopic species is also recommended in studying the impact of human activities on arthropod communities (Samways 1993, New 1995, Dufrêne and Legendre 1997). In this study, the target species were halophilic species, defined by their preference or exclusive presence in salt-marsh habitats (Kamer et al. 2008), which can be assessed using distribution maps (in our study, relevant atlases are Luff 1998 and Turin 2000). Two approaches were used for assessing changes in natural salt marshes compared to invaded ones: (i) a diachronic (before vs. after the invasion) comparison of species distribution along a land-sea gradient and (ii) a synchronic comparison of species assemblages between invaded and natural habitats.

## Methods

### Study site and sampling design

**Figure 1.**
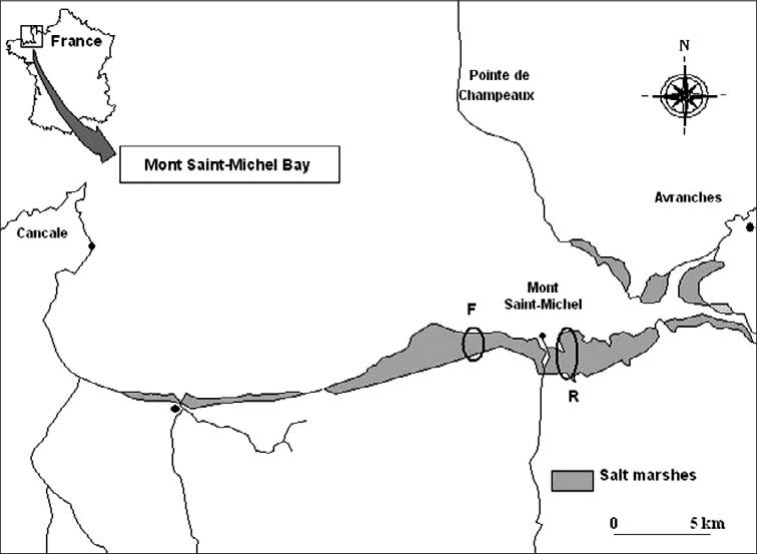
Location of the study sites (Mont St-Michel Bay, France). Codes: **F** ‘Ferme Foucault’ **R** ‘la Rive’.

The Mont Saint-Michel bay (NW France) is an extensive littoral zone (500 km²) located between the regions Brittany and Normandy (48°40’N, 1°40’W). Two sites have been studied in salt marshes: “la Ferme Foucault”, on the western part of the Mont St.-Michel (coded F; 48°37’N, 1°32’W) and “la Rive” on the eastern part of the Mont St.-Michel (coded R; 48°37’N, 1°29’W) (Fig. 1).

For the diachronic approach, ground beetle populations were compared at seven stations (A to G) located along the same land-sea transect at the “Ferme Foucault” site between 1983–1984 and 2002. During the study of 1983–1984, *Elymus athericus* was restricted in this salt marsh to the dyke (station A) and to the upper marsh (station B), but absent from stations C-G. Invasion by *Elymus athericus* modified the plant cover of the sampling stations between 1984 and 2002. The middle marsh and lower marsh stations (station C till F), dominated in 1984 by *Atriplex portulacoides* (Chenopodiaceae), were dominated by *Elymus athericus* in 2002.

Secondly, natural (dominated by *Atriplex portulacoides*), and invaded (dominated by *Elymus athericus*) stations were studied at different marsh levels in the synchronic approach. Comparisons of paired stations (natural and invaded – coded N and I, respectively) were spatially replicated three times for avoiding pseudo-replication (Hulbert 1984). Paired stations were located at the same distance from the dyke because of the existence of a salinity gradient influencing both species richness and abundance (Pétillon et al. 2004): stations 1 (350m), stations 2 (800–900m; both couples of stations at the “Ferme Foucault” site) and stations 3 (1000 meters from the dyke; “La Rive” site). Because of the clonal progression of the invasive species, all *Elymus* populations (stations I1, I2 and I3) formed a uniform and continuous plant cover. The natural areas sampled were either patch-like formations (in case of stations N1 and N2) or strip-like formations (station N3). Mean salinities did not significantly differ between invaded and natural stations at each salt marsh level (Pétillon et al. 2005) and elevations were similar between compared stations (J.C. Castel and J. Huet, 1999, unpublished data). More details on the sampling stations can be found in Fouillet (1986) and Pétillon (2005).

### Sampling techniques and species identification

For both the synchronic and diachronic approaches, ground beetles were sampled with pitfall traps, consisting of polypropylene cups (10 cm diameter, 17 cm deep) with ethylene-glycol as preservative. Traps were covered with a raised wooden roof to keep out rain. They were emptied weekly when tides permitted (i.e., about three weeks per month). Pitfall traps were grouped by four and spaced 10 m apart, this being considered to be the minimum distance for avoiding interference between traps (Topping and Sunderland 1992). Before the *Elymus* invasion, Fouillet (1986) sampled the transect with one trap per station from May to September in 1983 and 1984, for a total of 16 five-day samples. In 2002, four traps were installed at each station in both study sites, from April to November 2002. Sampling time was comparable between both periods (90 days in 1983–1984 and 96 days in 2002). Because of the differences in sampling efforts, we only compared the two studies on the basis of species presence / absence (i.e., distribution range along the land-sea transect).

Ground beetles were preserved in 70% ethanol and identified using Jeannel (1942) and Trautner and Geigenmüller (1987). Nomenclature follows Lindroth (1992) as far as possible, and Fauna Europaea otherwise (http://www.faunaeur.org/).

### Data analyses

Statistics on the abundances of halophilic species were performed only for species represented by at least 10 individuals in couples of stations. Catches in pitfall traps were related to trapping duration and pitfall trap perimeter, which calculates an “activity trappability density” (number of individuals per day and per meter – Sunderland et al. 1995). Mean species richness and mean abundances were compared using a two-way mixed model (habitat × station) with habitat type as fixed factor, station (1, 2 and 3) and interaction habitat*station as random factors. In case of non-significant interaction between habitat type and station, the interaction was removed from the model and a new model was performed for detecting significant effects of habitat type and/or station. In case of significant interaction between habitat type and station, parameters were analysed station by station (one-way ANOVA). Statistical analyses were performed using the Statistica-7 software.

## Results

### Diachronic approach

A total of 24 species (represented by 7,774 individuals) and 35 species (represented by 8,588 individuals) were caught in 1983–1984 and in 2002, respectively. Five species were exclusive to the first sampling period and 16 to the second one. All the species that were only recorded in 1983–1984 were caught in very low numbers (max. 2 individuals), four species on the dyke (*Clivina colaris*, *Dromius linearis*, *Harpalus rufibarbis* and *Harpalus rufipes*) and only one in the salt marsh (*Dyschirius chalceus*). As the sampling effort was quite different between 1983–1984 and 2002 (see Material and Methods), it cannot be concluded that the ‘appearance’ of species between the two studies can be related to the invasion by *Elymus athericus*. The comparison in distribution was thus restricted to the 19 shared species (Table 1).

In terms of distribution ranges, two groups of carabids were distinguished: species with constant distribution range in the salt marsh or on the dyke and species with an increased distribution range between 1983–1984 and 2002. Eight species were caught on the dyke in 1983–1984 and in 2002, and seemed not to have progressed with *Elymus athericus* in the salt marsh (*Amara equestris*, *Anisodactylus binotatus*, *Bembidion tetracolum*, *Harpalus anxius*, *Leistus fulvibarbis*, *Nebria brevicollis*, *Pterostichus melanarius* and *Pterostichus niger*: Table 1). Eight other species had a similar habitat range in the salt marsh, extending from the upper to lower marsh or from the dyke to the lower marsh (halophilic species: bold in Table 1), plus two high-marsh living species (*Badister bipustulatus* and *Pterostichus vernalis*), one low-marsh living species (*Dyschirius salinus*) and one species with a discontinuous range along the land-sea transect (*Loricera pilicornis*).Only three speciesshowed an extension of their distribution in the salt marsh, both to the upper and lower marsh (*Bembidion iricolor*, *Bembidion lampros* and *Pterostichus cupreus*).

**Figure 2.**
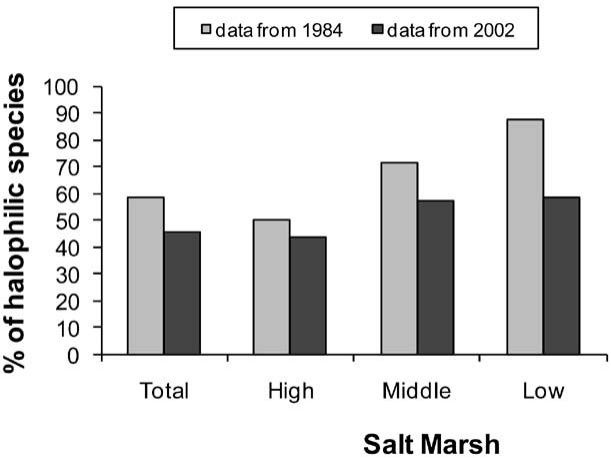
Changes in the percentage of halophilic species in the salt marsh after the invasion by *Elymus athericus*.

The *Elymus athericus* invasion led to a decrease in the percentage of halophilic species in invaded salt marshes (Fig. 2).

**Table 1.** Comparison of total catches (number of individuals) between 1983–1984 and 2002 along a land-sea transect (Foucault site; bold: halophilic species). The letters **A–G** indicate different sampling stations. In 1983–1984, only stations **A–B** had a dominant *Elymus athericus* cover; in 2002 at all stations *Elymus athericus* was present (dominant cover for stations **A** to **F**).

	*Period*	*A*	*B*	*C*	*D*	*E*	*F*	*G*	*Total*
*SHARED SPECIES*									
*Amara equestris*(Duftschmid 1812)	1983–84	1							1
	2002	1							1
*Anisodactylus binotatus*(Fabricius 1787)	1983–84	3							3
	2002	6							6
*Badister bipustulatus*(Fabricius 1792)	1983–84		1						1
	2002	2	2						4
*Bembidion iricolor*Bedel 1879	1983–84	2		2	2				6
	2002	5	98	89	21	1	4		218
*Bembidion lampros*(Herbst 1784)	1983–84	1		2					3
	2002	1	12	18	3	5	4	1	44
*Bembidion minimum* (Fabricius 1792)	1983–84	4	1	40	52	10	2		109
	2002	1	31	13	5	80	96	3	229
*Bembidion normanum* Dejean 1831	1983–84	1		24	39	244	149	53	510
	2002	2	6	8	13	212	131	24	396
*Bembidion tetracolum* (Say 1823)	1983–84	1							1
	2002	1							1
*Dicheirotrichus gustavii* Crotch 1871	1983–84	2		11	83	2121	2622	393	5232
	2002	2	8	2	3	136	237	156	544
*Dyschirius salinus* Schaum 1843	1983–84						1	1	2
	2002							5	5
*Harpalus anxius*(Duftschmid 1812)	1983–84	2							2
	2002	1							1
*Leistus fulvibarbis* Dejean 1826	1983–84	3							3
	2002	1							1
*Loricera pilicornis*(Fabricius 1775)	1983–84				1				1
	2002					1		1	2
*Nebria brevicollis* (Fabricius 1792)	1983–84	3							3
	2002	1							1
*Pogonus chalceus* (Marsham 1802)	1983–84	8	4	65	42	678	617	436	1850
	2002	13	100	193	126	1628	1290	2243	5593
*Pterostichus cupreus*(Linnaeus 1758)	1983–84	3		5					8
	2002	7	41	9			2		59
*Pterostichus niger*(Schaller 1783)	1983–84	24							24
	2002	1							1
*Pterostichus vernalis*(Panzer 1795)	1983–84	4							4
	2002	2	1						3
*Pterostichus melanarius*(Illiger 1798)	1983–84	4							4
	2002	12							12
*SPECIES NOT RECOLLECTED IN 2002*									
*Clivina collaris*(Herbst 1786)	1983–84	2							2
	2002								0
*Dromius linearis*(Olivier 1795)	1983–84	1							1
	2002								0
*Dyschirius chalceus* Erichson 1837	1983–84							1	1
	2002								0
*Harpalus rufibarbis*(Fabricius 1792)	1983–84	2							2
	2002								0
*Harpalus rufipes*(Degeer 1774)	1983–84	1							1
	2002								0
*NEW SPECIES FOUND IN 2002*									
*Anchomenus dorsalis*(Pontoppidan 1763)	1983–84								0
	2002	1							1
*Agonum muelleri*(Herbst 1784)	1983–84								0
	2002			5	1				6
*Amara lunicollis*Schiödte 1837	1983–84								0
	2002	3							3
*Amara plebeja*(Gyllenhal 1810)	1983–84								0
	2002		2					1	3
*Amara tibialis*(Paykull 1798)	1983–84								0
	2002	2							2
*Anisodactylus poeciloides* (Stephens 1828)	1983–84								0
	2002			2					2
*Bembidion obtusum*Serville 1821	1983–84								0
	2002	10	16	1					27
*Calathus mollis*(Marsham 1802)	1983–84								0
	2002		1						1
*Clivina fossor*(Linnaeus 1758)	1983–84								0
	2002							1	1
*Dicheirotrichus obsoletus* (Dejean 1829)	1983–84								0
	2002		2	12	5	478	572	301	1370
*Harpalus distinguendus*(Duftschmid 1812)	1983–84								0
	2002	1		2					3
*Harpalus melancholichus*Dejean 1829	1983–84								0
	2002	1							1
*Microlestes minutulus*(Goeze 1777)	1983–84								0
	2002	2							2
*Pogonus littoralis* (Duftschmid 1812)	1983–84								0
	2002	1	13		1				15
*Pterostichus versicolor*(Sturm 1824)	1983–84								0
	2002	4	14	2					20
*Tachys scutellaris* Stephens 1828	1983–84								0
	2002		10						10
*Total*		*156*	*363*	*505*	*397*	*5594*	*5727*	*3620*	*16362*

### Synchronic approach

A total of 505 individuals belonging to 17 species were sampled in the three pairs of natural and invaded stations. The synchronous comparison of natural and invaded habitats revealed the existence of eight shared species. Two species were exclusive to natural habitats (*Pogonus littoralis* and *Pogonus luridipennis*) and six to invaded habitats (*Anisodactylus poeciloides*, *Bembidion obtusum*, *Harpalus anxius*, *Harpalus distinguendus*, *Pterostichus cupreus* and *Pterostichus versicolor*). Total species richness was higher in invaded habitats than in the natural ones (Table 2). Significant interactions between habitat type and station were found for species richness and two species *Pogonus chalceus* and *Dicheirotrichus gustavii*. Mean species richness was significantly higher in an invaded station compared to its adjacent natural one (one-way Anova, F-ratio=22.04, p=0.003, d.f.=7). More *Pterostichus chalceus* were caught at a natural station than at the paired invaded one (one-way Anova, F-ratio=14.68, p=0.009, d.f.=7). *Dicheirotrichus gustavii* was significantly higher in an invaded station compared to the natural one (one-way Anova, F-ratio=6.89, p=0.039, d.f.=7) and the opposite pattern was found in another couple of stations (one-way Anova, F-ratio=11.94, p=0.014, d.f.=7). *Bembidion minimum* was significantly higher in invaded habitats compared to natural ones (Factorial Anova, F-ratio=5.91, p=0.025, d.f.=20). No difference between habitat types was found for *Dicheirotrichus obsoletus* and *Bembidion normanum*(Table 2).

**Table 2.** Comparison of total species richness (total S), mean species richness (mean S) and means abundances (as expressed in number of individuals per day and per meter) of *Pogonus chalceus*, *Dicheirotrichus obsoletus*, *Bembidion normanum*, *Dicheirotrichus gustavii* and *Bembidion minimum* between natural (**N**) and invaded (**I**) habitats. Means in bold are significantly different (p<0.05) between habitat types (mean ± s.e., see text for details in statistics).

*Total S*	*N*	*I*	*N1*	*I1*	*N2*	*I2*	*N3*	*I3*
	*11*	*14*						
Mean S	6.17±0.35	6.92±0.51	*5.50±0.50*	*8.75±0.48*	6.25±0.63	6.50±0.65	6.75±0.63	5.50±0.65
*Pogonus chalceus*	7.66±1.90	4.75±1.91	1.11±0.32	1.68±0.24	13.50±3.35	10.76±4.63	*8.37±1.55*	*1.80±0.74*
*Dicheirotrichus obsoletus*	1.38±0.60	1.68±0.72						
*Bembidion normanum*	0.78±0.35	0.98±0.40						
*Dicheirotrichus gustavii*	0.45±0.15	0.68±0.29	0.03±0.02	0.02±0.01	*1.14±0.15*	*1.99±0.29*	*0.19±0.04*	*0.05±0.02*
*Bembidion minimum*	*0.29±0.11*	*0.52±0.10*						

## Discussion

By comparing data from 1983–1984 to 2002, we could show that only three species have extended their distribution range with the *Elymus* invasion, despite the existence of several dyke-inhabiting species (eight continental species with constant distribution). This result is opposite to those obtained for spiders in the same study site, with many range-expanding species (Pétillon et al. 2005). This pattern can also be related to the high percentage of halophilic carabid species found in salt marshes, much higher than for spiders (Pétillon et al. 2008). Assemblages of ground beetles in salt marshes proportionally contain more specific, halophilic species, and continental species are conversely unlikely to colonize this habitat. Meijer (1980) also noted that spiders were less sensitive to variations in soil salinity than ground beetles. Higher percentages of stenotopic species in ground beetle assemblages than in spider assemblages have been recorded in other flooded habitats, such as river floodplains (Rothenbücher and Schaefer 2006) and riverbanks (Bonn and Kleinwächter 1999).

Although the sampling effort was quite different between 1983–1984 and 2002, we assume that around 11 records of the 16 new species during the second sampling period can also be due to the invasion by *Elymus*. In fact, several continental species were discovered after the invasion in relatively high numbers (i.e., more than five individuals), both on the dyke and in the salt marsh. Among them, most species are linked to high contents of organic matter and a more pronounced litter layer (e.g., *Agonum muelleri*, *Bembidion obtusum* and the polyphagous *Pterostichus versicolor*) or are even partly phytophagous (*Amara* spp. and *Harpalus*spp.: Dajoz 1988, Ikeda et al. in press). Conversely, halophilic species discovered in 2002 are hardly related to the invasion. *Pogonus littoralis* and *Dicheirotrichus obsoletus* could have been misidentified earlier, as these species are very similar to *Pogonus chalceus* and *Dicheirotrichus gustavii*, respectively (Forel and Leplat 2005, Dhuyvetter et al. 2007). *Dicheirotrichus obsoletus* could also have been missed in 1983–1984 (the sampling stopped in September) as more than 89% of individuals were caught in October-November during 2002. *Tachys scutellaris* appears as a new species in 2002, but was present in 1983–1984, but at another station located below the mean sea level (slikke habitat: Fouillet 1986). The ‘appearance’ of several species, sampled in low numbers in 2002, can be due to differences in sampling effort and/or to random catches.

The synchronic study revealed that almost half of the species (8/19), both continental and halophilic ones, were shared between natural and invaded habitats. Three species, all halophilic, were exclusive to natural habitats. Conversely, six species were exclusive to invaded habitats, among them some of the species that colonized the marsh after the invasion by *Elymus athericus* (e.g., *Bembidion lampros* or *Pterostichus cupreus*). New conditions created by the grass *Elymus* – mainly an enhanced litter layer and higher plant cover – thus lead to the establishment of several continental species directly or indirectly linked to organic matter or to the litter (as shown by Pétillon et al. 2008).

Although few deleterious impacts of invasion by *Elymus athericus* on carabids were found, management could be necessary to reduce the effects of invasion and decrease the rate of spread of the invasive plant. Sheep grazing – despite being a good potential method for biological control of invaders (Shea and Chesson 2002) – is at the moment carried out too intensively in the Mont Saint-Michel bay, leading to a decrease in carabid species richness (Pétillon et al. 2007). A low stocking rate (i.e., between 0.5 and 1.5 sheep ha-1) can therefore be recommended, assuming greatest positive effects at intermediate disturbance intensities (for arthropods: e.g., Dennis et al. 2001, Suominen et al. 2003).

Long-term monitoring of population dynamics is thus recommended for halophilic species in invaded, natural and managed habitats. Special attention could be paid to less dominant species, as their small populations could be reduced faster than other, dominant, salt-marsh carabids. This study confirms the high value of carabids as bioindicators (as they present a high percentage of specialist species) and shows the possibility of using long-term surveys for ecological studies, if carefully interpreted.
